# Molecular Basis of Late-Life Depression

**DOI:** 10.3390/ijms22147421

**Published:** 2021-07-10

**Authors:** Chien-Yi Kuo, Chieh-Hsin Lin, Hsien-Yuan Lane

**Affiliations:** 1 Department of Psychiatry, China Medical University Hospital, Taichung 40402, Taiwan; sharron_ky@hotmail.com; 2 Graduate Institute of Biomedical Sciences, China Medical University, Taichung 40402, Taiwan; 3 Department of Psychiatry, Kaohsiung Chang Gung Memorial Hospital, Chang Gung University College of Medicine, Kaohsiung 83301, Taiwan; 4 School of Medicine, Chang Gung University, Taoyuan 33302, Taiwan; 5 Department of Psychology, College of Medical and Health Sciences, Asia University, Taichung 41354, Taiwan

**Keywords:** late-life depression, glutamatergic system, NMDA receptor, IL-6, cytokine, BDNF

## Abstract

Late-life depression (LLD), compared to depression at a young age, is more likely to have poor prognosis and high risk of progression to dementia. A recent systemic review and meta-analysis of the present antidepressants for LLD showed that the treatment response rate was 48% and the remission rate was only 33.7%, thus implying the need to improve the treatment with other approaches in the future. Recently, agents modulating the glutamatergic system have been tested for mental disorders such as schizophrenia, dementia, and depressive disorder. Ketamine, a noncompetitive NMDA receptor (NMDAR) antagonist, requires more evidence from randomized clinical trials (RCTs) to prove its efficacy and safety in treating LLD. The metabotropic receptors (mGluRs) of the glutamatergic system are family G-protein-coupled receptors, and inhibition of the Group II mGluRs subtypes (mGlu2 and mGlu3) was found to be as effective as ketamine in exerting rapid antidepressant activity in some animal studies. Inflammation has been thought to contribute to depression for a long time. The cytokine levels not only increase with age but also decrease serotonin. Regarding LLD, interleukin 6 (IL-6) and tumor necrosis factor α (TNF-α) released in vivo are likely to contribute to the reduced serotonin level. Brain-derived neurotrophic factor (BDNF), a growth factor and a modulator in the tropomyosin receptor kinase (Trk) family of tyrosine kinase receptors, probably declines quantitatively with age. Recent studies suggest that BDNF/TrkB decrement may contribute to learning deficits and memory impairment. In the process of aging, physiological changes in combination with geriatric diseases such as vascular diseases result in poorer prognosis of LLD in comparison with that of young-age depression. Treatments with present antidepressants have been generally unsatisfactory. Novel treatments such as anti-inflammatory agents or NMDAR agonists/antagonists require more studies in LLD. Last but not least, LLD and dementia, which share common pathways and interrelate reciprocally, are a great concern. If it is possible to enhance the treatment of LDD, dementia can be prevented or delated.

## 1. Introduction

Aging is a global trend. The UN statistics shows that the population over age 65 by 2050 will reach 16% of total population. (https://www.un.org/en/global-issues/ageing?fbclid=IwAR2ut7ufS5ULfFGf4HbXtijNmx2q0VFzzIyBy0Fonznzt87LeIMjJGK21nU (accessed on 9 July 2021). Depression has recently been identified as a pandemic, causing huge social cost and high financial load. The average prevalence of geriatric depression in the community is about 12%, and, in long-term care institutions, 35% of patients have significant depressive symptoms [1].

Unlike depression at a young age, late-life depression (LLD), with high pathogenic complexity caused by physiological and psychosocial issues and chronic disease, can rarely be treated with one antidepressant. A recent systemic review and meta-analysis of the present antidepressants for LLD showed that the treatment response rate and the depression remission rate are 48% and 33.7% [2], respectively. It is thought that biological factors are pathogenically associated with LLD [3]. In the process of aging, structural changes in the brain may be associated with depression. The causes of LLD may include not only neuroendocrine dysregulation and changes in neural circuitry, but also genetic vulnerability and stress due to life events that interact reciprocally [4,5].

## 2. Depression and Late-Life Depression

According to the 2016 CANMAT guideline, the first-line drug recommendations for adult depression include SSRIs, SNRIs, agomelatine, bupropion, and mirtazapine [6]. For elderly depression, the first-line drug recommendations include duloxetine, mirtazapine, and nortriptyline (level of evidence: level 1), as well as bupropion, citalopram/escitalopram, desvenlafaxine, duloxetine, sertraline, venlafaxine, and vortioxetine (level of evidence: level 2) [7].

In two large-scale studies, IMPACT [8,9] and PROSPECT [10,11] trials for elderly depressed patients, there was a difference in response rate between receiving antidepressant treatment and receiving general care only. The results of the IMPACT and PROSPECT trials showed that the antidepressant group had a better response rate (IMPACT, antidepressant vs. normal care: 45% vs. 19%; PROSPECT, antidepressant vs. usual care: 43% vs. 28%). Compared with young depression, late-life depression displays more somatic symptoms and cognitive deficits. The somatic symptoms include hypochondriasis, general and gastrointestinal somatic symptoms, and agitation [12,13,14]. Moreover, old age may be accompanied by other chronic diseases. When somatic complaints occur, depression may not be the first diagnosis, thus leading to underdiagnosis [15]. In the past, meta-analyses found different response rates of antidepressants between different groups: 53.8% in early-onset depression vs. 44.4% in late-onset depression [16,17]. Coupled with the analysis of subgroups, it was found that depressed people younger than 55 years had a significantly higher response rate to antidepressants than those older than 65 years. In addition, there was no significant difference between the group older than 65 and the placebo group. In the STAR*D trial [18], the overall remission rate in the acute treatment step was 67%. With more treatment steps, the remission rate continued to decrease, from 36.8% in the first step to 13% in the fourth step. Approximately 50% of patients will develop treatment resistance to antidepressants over time. Some studies found that treatment resistance for first-line antidepressants in elderly patients was as high as 55–81% [19]. Some studies suggest that lithium may be effective for treatment-resistant late-life depression [20,21]; however, more replicative studies are warranted. Esketamine is a recent FDA-approved treatment for treatment-resistant depression, but its efficacy and safety in the elderly have not yet been confirmed. A recent phase 3 clinical trial [22] enrolled treatment-resistant depression patients over 65 years old and randomly assigned them to the nasal spray esketamine/antidepressant or nasal spray placebo/antidepressant group for 4 weeks. According to the change in scores of MADRS as the primary endpoint, there were no significant differences between both groups.

## 3. Late-Life Depression and Suicide

The average prevalence of geriatric depression in the community is about 12%, and, in long-term care institutions, 35% of patients have significant depressive symptoms [1]. According to previous studies [23,24], the incidence of depression in women was relatively high. Regarding suicide, the methods used by the elderly are usually more lethal than those used by the young [25]. Studies have also found that depression and suicide are strongly correlated. Of course, depression symptoms themselves are not the single cause of suicide. However, the overall suicide rate of people taking antidepressants seems to be relatively low [26]. A recent American study [27] enrolled 225 elderly people with an average age of 71.4 years who were eligible for the diagnosis of depression. It was found that low education, being male, and recent stressful events were strongly associated with suicidal ideation, especially recent stressful events (*p* < 0.001). A study in the United Kingdom [28], which analyzed the characteristics of early-onset depression and late-onset depression suicides, found that psychiatric hospitalizations were rare among people with late-onset depression and other psychiatric comorbid diagnoses. Of note, there were more life stress events before suicide.

## 4. Depression and Dementia

Late-life depression is usually considered a chronic course, accompanied by cognitive impairment. Depression is considered to be one of the risk factors of dementia or a prodrome of dementia. Research [29] suggests that late-life depression may increase dementia risk by twofold. A recent meta-analysis [30] showed that depression in later years is associated with dementia in all forms. Further analysis discovered that the risk (2.52, 95% CI 1.77–3.59, *p* < 0.001) of vascular dementia is higher than Alzheimer`s disease (1.65, 95% CI 1.42–1.92, *p* < 0.001).

The two seem to overlap in some neurobiological findings. The theory of the HPA axis is currently the most consistent in depression research. Elevated cortisol level can also cause hippocampus neuronal loss and volume reduction [31,32]. Hypercortisolemia can also be seen in the CSF of patients with Alzheimer’s disease (AD) [33]. Vascular depression refers to the appearance or aggravation of depression after the occurrence of cerebrovascular events. In addition to WMHs seen on neuroimaging, there are lesions on small blood vessels (maybe subcortical infarcts, microbleeding, etc.). In addition, in cases of depression, decreased blood flow in the brain may also lead to hyperactivity of the hippocampus and amygdala [34]. Inflammation can be seen in people with AD and depression, due to an increase in activated microglia in the CNS [35,36]. Microglial cells that are continuously activated have a reduced ability to remove neurotoxic agents, leading to a reduction in neuronal loss and neurogenesis [34]. An increase in peripheral proinflammatory markers is also associated with the severity of depressive symptoms and cognitive impairment [37]. Neurotrophic factors include BDNF, the main function of which is to regulate synaptic plasticity, which plays an important role in learning and memory. It has been found in patients with depression that the use of antidepressants can increase the concentration of BDNF in the blood [30,38]. In patients with AD, the severity of cognitive impairment is related to BDNF and amyloid beta (Aβ1–42) plasma levels in serum [39,40]. Although the current research has found some common neurobiological changes in depression and AD, there are overlaps in symptoms. However, according to the current research, there is insufficient evidence that antidepressants can improve the cognition of depression in the elderly and the depression symptoms of AD [41,42,43,44].

## 5. Mechanisms Underlying Depression/Late-Life Depression

### 5.1. Glutamatergic System

Glutamatergic synapses are excitatory synapses that are associated with cerebral regions related to depression and stress, such as the prefrontal cortex (PFC), hippocampus, and amygdale. Glutamate receptors belong to two groups: ionotropic receptors and G-protein coupled metabotropic receptors (mGluRs); the former contains three subgroups: *N*-methyl-d-aspartate (NMDA), α-amino-3-hydroxy-5-methyl-4-isoxazolepropionic acid (AMPA), and kainite receptors. The NMDA receptor formed by two GluN1 subunits and two GluN2 subunits is a tetrameric glutamate with ligand-gated and voltage-gated ion channels. Ligand-gated channels are only activated when the co-agonist (e.g., glycine or d-serine) and the agonist (e.g., glutamate) bind to it concurrently [45]. Ca^2+^ in cell conduction mainly serves as a second messenger; it is associated with gene regulation, excitement of the cell membrane, and synaptic plasticity. In the past, aging was thought to be related to changes in Ca^2+^ regulation [46]. NMDA receptors possess a Ca^2+^ channel that triggers subsequent message conduction through the influx flow of Ca^2+^. The GluN1 subunit of the NMDA receptor is mainly distributed in the hippocampus, and, during the aging process, the GluN1 protein levels in the hippocampus decrease significantly [47,48]. The GluN2B subunit’s calcium ion channel flow rate is slower and it is slower to close, which has a large impact on the overall Ca^2+^ fluidity; thus, it is considered to have a greater impact on synaptic activity. The upregulation of GluN2B may enhance the role of LTP, thereby improving learning and memory [49].

Ketamine, a noncompetitive NMDA receptor antagonist, is the most clinically and empirically proven glutamate agent for treatment-resistant depression, as a rapid-acting antidepressant. While the mechanism of ketamine in the brain is not fully understood, some studies have shown that it increases the AMPA/NMDA receptor activity ratio by blocking the NMDA receptor first and activating the AMPA receptor later, subsequently activating mammalian target of rapamycin (mTOR) signaling by releasing BDNF [50,51]. In addition to (*S*)-ketamine, which was approved by US FDA and marketed in 2019, the enantiomers (*R*)-ketamine and (*S*)-norketamine also have therapeutic potential. In animal studies, (*R*)-ketamine leads to fewer behavioral abnormalities and a more extended antidepressant effect. The (*S*)-enantiomer may induce psychosis by decreasing the binding availability of dopamine receptors and increasing presynaptic dopamine release [52]. In fact, the pathways for LLD are much more complex. For example, other NMDA-related biomarkers and even d-aspartate are also involved. A change in the peripheral mRNA expression levels of NMDAR genes was found in individuals with MDD [53]. Not only antagonists, but also agonists of NMDAR show antidepressant efficacy. d-Serine is a co-agonist of NMDAR, and, in a single-dose administration of 2.1 g orally, it led to a reduction in subjective feelings of sadness and anxiety in the healthy group [54]. It seems that d-serine and ketamine share the common pathway mentioned above [55]. d-Serine also showed the potential to improve cognitive function in some animal studies, through exogenous d-serine supplementation [54,56]. Sodium benzoate is a d-amino-acid oxidase inhibitor, which can block the metabolism of d-serine and increase its levels. Research has shown that sodium benzoate can improve cognitive impairment in early-phase AD and in schizophrenia [57,58]. Even in late-phase AD, there was a significant difference in cognitive improvement between benzoate treatment group and placebo. Additionally, the results showed female preference [59]. A recent study also demonstrated that sodium benzoate can lead to an improvement in brain activity and cognitive function in MCI [60]. Declining cortical GABA concentrations [61,62,63] and abnormal NMDA receptor levels [63,64] in the aging brain and in patients with depression at the age of 60 and over imply the role of ketamine in treating LLD. Ketamine’s safety and validity as an antidepressant for LLD were proven by a few small double-blind [65], randomized, and active placebo-controlled trials; however, a huge RCT is required as a robust piece of evidence in the future.

Memantine, as another noncompetitive NMDA receptor antagonist, was proven to enhance the BDNF level [66] and inhibit depressive behaviors in animal trials. The pilot study [67] assessed the effects of a combined treatment of antidepressants and memantine for LLD and impaired cognition with participants aged 50–90 recruited from the outpatient department of the Late Life Depression Clinic and the Memory Disorders Clinic at the New York State Psychiatric Institute and the Behavioral Neurologists’ practice group at Columbia University Medical Center, who received neuropsychological testing and remitted depression assessment at baseline and at weeks 12, 24, and 48. The escitalopram intake was 10 mg/day during week 1 and 20 mg/day for the remainder of the study. The maximal intake was either 20 mg/day or the tolerable daily intake. The memantine intake was 5 mg/day during week 2, with the intake hiked on a weekly basis, such that the maximal intake was either 20 mg/day in week 6 or the tolerable daily intake. The subjects consisted of 35 elderly cases with depression and cognitive impairment with the escitalopram mean daily dose of 18.62 mg (SD 5.15) and the memantine mean daily dose of 13.62 mg (SD 6.67). The results showed an improvement in Hamilton Depression Rating Scale scores and in cognitive functions (*p* = 0.0147). A recent RCT study [68] (a 6 month double-blind placebo-controlled trial) compared the effects of escitalopram + memantine (ESC + MEM) and escitalopram + placebo (ESC + PBO) for LLD and incident subjective memory complaints, where the samples with subjective memory complaints were prone to mild cognitive impairment and Alzheimer’s disease. Prior to the trial, the subjects suspended personal psychotropic medications for more than 2 weeks and fluoxetine for more than 4 weeks. As a randomized clinical trial, the subjects consisted of 95 cases randomly distributed to the ESC + MEM/ESC + PBO groups with an escitalopram daily intake of 10–20 mg/day in both groups and a memantine daily intake of 5 mg/day initially and 10 mg twice/day later on in the ESC + MEM group. The remission rate of LLD was the primary outcome. The definition was an HAM-D score of no more than 6. The samples in both groups received an assessment in month 3 and month 6. The remission rates of the ESC + MEM and ESC + PBO groups in months 3 and 6 were 45.8% and 47.9% and 38.3% and 31.9%, respectively (*p* = 0.15). Both groups failed to present a significant difference in the tolerance and in the dropout rate; however, the ESC + MEM group presented a higher improvement.

**Figure 1:**
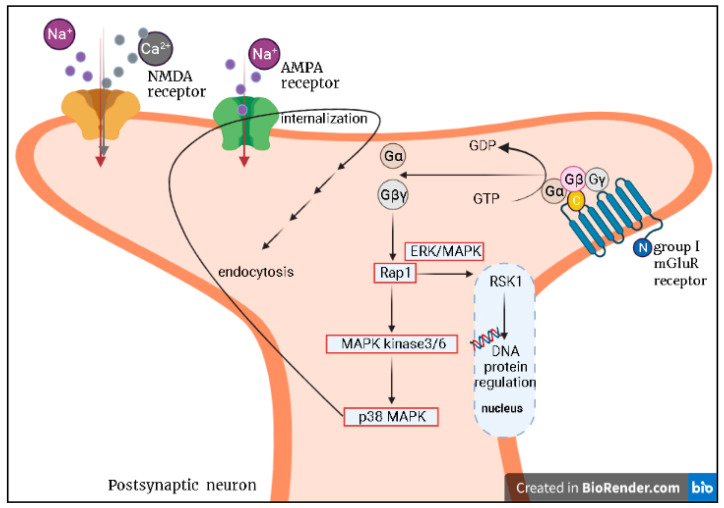
Signal pathway involved in mGluR–LTD. Metabotropic receptors (mGluRs) are G protein-coupled receptors. G proteins are activated when GTP is converted to GDP, and the three subunits (α, β, and γ) are dissociated. The release of G β and γ subunits activates Rap 1 and MAPK kinase 3/6. Subsequently, P38 MAPK, after it is activated, promotes AMPA receptor internalization and endocytosis. Additionally, the Rap 1–MAPK/ERK pathway activates nuclear protein RSK1. MAPK: mitogen-activated protein kinases; ERK: extracellular signal-regulated kinases; RSK1: ribosomal S6 kinase-1.

Metabotropic receptors (mGluRs) represent nonionotropic, G-protein-coupled receptors in the glutamatergic system which can be categorized into three subgroups according to their sequence similarities, pharmacological properties, and intracellular signal transduction mechanisms: group I including mGluR1 and mGluR5, group II including mGluR2 and mGluR3, and group III including mGluR4, mGluR6, mGluR7, and mGluR8 [69,70]. Synaptic plasticity refers to the ability of synapses to strengthen or weaken over time in response to increases or decreases in their activity, which is thought to be associated with learning and memory. Long-term potentiation (LTP) is induced by high-frequency electrical stimulation to glutamatergic synapses. Previous studies focused on NMDARs as the essential mediators in long-term memory formation, with overactive NMDARs considered neurotoxic. Long-term depression (LTD) is modulated by synaptic NMDARs (NMDAR-LTD) or by mGluRs (mGluR-LTD). It is thought that solitary stimulation of group I mGluRs (mGluR1 and mGluR5) is able to induce LTD, while synaptic AMPA receptors generate endocytosis (Figure 1) [71,72]. Dysregulation of mGluR–LTD contributes to both learning deficits and neuropathological conditions such as fragile X syndrome, mental retardation, and Alzheimer’s disease [73]. The metabotropic glutamate receptors (mGluRs) are thought to contribute to neurological and psychiatric disorders, including major depressive disorder [74]. Activating the group II metabotropic glutamate receptor subtypes (mGlu2 and mGlu3) inhibits spontaneous excitatory neurotransmission [75,76,77] and induces postsynaptic LTD [78,79]. In a preclinical study [80], the mGlu2 and mGlu3 antagonist (1*S*,2*R*,3*S*,4*S*,5*R*,6*R*)-2-amino-3-[(3,4-difluorophenyl)-sulfanylmethyl]-4-hydroxy-bicyclo[3.1.0]hexane-2,6-dicarboxylic-acid (LY3020 371) was used to inhibit the mGlu2/3 receptors in the cortex and hippocampus of rats/mice. It was proven to be a valid antidepressant as good as ketamine. Among the agents modulating the glutamatergic system, ketamine is widely used and publicly known for treatment-resistant depression. In the future, mGlu2/3 receptor agents as a single or combined drug with ketamine or other antidepressants can likely be used to exert significant effects for depression, including for the elderly. Additionally, NMDARs and metabotropic glutamate receptors (mGluRs) are associated with the modulation of long-term potentiation (LTP) and long-term depression (LTD) (Figure 1). Depression is a risk factor of dementia. It is possible to reduce the family care load and huge social cost linked to remitted depression, which can lower dementia risk, by treating cognitive impairment via modulation of the LTP and LTD strengths.

### 5.2. Hypothalamic–Pituitary–Adrenal (HPA) Axis and Immunological Biomarkers

Depression is considered to be associated with acute and chronic stresses. Stress increases glutamate levels and modulates the hypothalamic–pituitary–adrenal (HPA) axis and epigenetic regulation. Increasing glutamate levels produces neurotoxicity, resulting in neuronal damage and loss. The HPA axis, under the stimulation of stress, causes the glucocorticoids levels to rise. As found in animal experiments, an increased level of glucocorticoids can lead to neuronal atrophy [81,82] in the prefrontal cortex and hippocampus. Stress can also cause decreased expression and function of the BDNF protein in the prefrontal cortex and the hippocampus, along with a decrease in the peripheral concentration [83,84,85]. The prefrontal cortex and hippocampus are two areas considered to be associated with depression. Decreases in neuronal atrophy in these two areas may cause depression and the impairment of learning and memory.

Recent evidence has shown the correlation of inflammation, the HPA axis, and depression. Inflammation and the HPA axis impact one another, while long-term cytokine exposure affects the activity of the glucocorticoid receptor. Glucocorticoid resistance causes HPA axis hyperactivity and increases inflammation [86,87]. The HPA axis function is regulated by glucocorticoid receptors, and epigenetic mechanisms such as DNA methylation can alter glucocorticoid receptors (GRs). In a recent study in South Korea [88], an attempt was made to analyze the correlation between GR DNA methylation and the incidence of depression in old age. A total of 732 local residents over 65 years were tracked, 521 of whom did not suffer depression at baseline. Blood was sampled from surrounding areas to analyze the GR gene, NR3C1, and the degree of DNA methylation in three different CpG sites; the results showed that the higher DNA methylation of CpG 2 was more related to the incidence of depression 2 years later. Microglia represent a brain-resident macrophage that can trigger innate immunity, and secreted BDNF plays a role in motor learning and memory, as well as the regulation of pain [89,90,91]. If microglia are active for a prolonged time period, the level of reactive oxygen species is increased in the brain, resulting in nerve cell death [92]. The production of an inflammatory brain reaction induces indoleamine 2,3-deoxygenate (IDO) upregulation, driving tryptophan toward the kynurenine pathway, leading to a decrease in serotonin production. Tryptophan is the precursor of serotonin. Tryptophan goes through two main metabolic pathways, with about 5% of tryptophan producing serotonin through the methoxyindole pathway, and about 95% going through the kynurenine pathway [93]. Tryptophan’s metabolized products via the kynurenine pathway are 3-hydroxykynurenine, hydroxyanthranilic acid, adenosine triphosphate (ATP), quinolinic acid, nicotinamide adenine dinucleotide, nicotinic acid, picolinic acid, KYN (kynurenic acid), indolepyruvic acid, indolelactic acid, and indoleacetic acid. The kynurenine pathway is mainly divided into two parts, extrahepatic and intrahepatic, with the majority of the pathway being carried out in the liver. The enzymes involved are different. In the liver, tryptophan 2,3-dioxygenase (TDO) is active, whereas the brain mainly involves indoleamine 2,3-dioxygenase (IDO), stored in astrocytes, microglia, and microvascular endothelial cells. When the body is exposed to stress or inflammation, TDO is activated and the IDO level is upregulated. Subsequently, tryptophan enters the kynurenine pathway, and the overall level of KYN increases, with exogeneous KYN entering the brain through the BBB [35,94]. Among this group of metabolites, KYN (kynurenic acid) can inhibit ionotropic glutamate receptors and attenuate glycerin co-agonist site activity on NMDA receptors. Quinolinic acid, an agonist of NMDA receptors, can inhibit the reuptake of glutamate by astrocytes, causing neurotoxicity-related [95] mental diseases such as depression. In addition, in suicide attempts, it was found that the concentration of quinolinic acid in CSF and plasma was increased [96].

A considerable number of studies have identified that interleukin-6 (IL-6) contributes substantially to major depressive disorder [97,98,99]. Not only does IL-6 reduce serotonin concentrations, but synaptic neuroplasticity is also impaired by interleukin-1β (IL-1β), which is released in vivo, contributing to cognitive impairment [100]. A high level of IL-6 was found to be related to increased risk for major depressive disorder (odds ratio = 2.49) in the elderly group in a longitudinal study [101] (Longitudinal Aging Study Amsterdam, LASA), independent of age, chronic disease, cognitive impairment, or use of antidepressants or anti-inflammation drugs. A 2 year South Korean [102] longitudinal study presented various correlations between five proinflammatory cytokine levels (TNF-α, IL-1α, IL-1β, IL-6, and IL-8) and late-life depression (LLD) from cross-sectional and prospective perspectives. The study collected 732 samples; 631 (82.6%) had no depression prior to the trial; 521 (521/631 = 0.826 = 82.6%) underwent a 2 year trace; 63 (63/521 = 0.12 = 12%) exhibited depression within a 2 year trace. Sex, cognitive function, disability, physical activity, and vascular risk score corrections and Bonferroni corrections were applied to the data collected. According to the findings, the IL-1β, IL6, and IL-8 levels of the 63 cases with incident depression increased; however, the comparative analytics among the IL-1β, IL-6, and IL-8 levels of the cases with incident depression at baseline and those with no incident depression at baseline failed to present a significant difference (χ^2^ = 0.544; *p*-value = 0.461). These outcomes imply that the cytokine levels are not an independent risk factor of incident depression. A recent study [103] presented how cytokines contribute to LLD and to cognitive impairment, i.e., learning deficits and memory impairment. The subjects consisted of 58 cases aged 60 and older, 24 with and 34 without depression. In accordance with the findings, the IL-1β, TNF-α, and IL-6 levels in the group with depression exceeded those without depression, and the high level of IL-6 contributed to memory impairment in the depressive group. These findings seem to conform to the findings of previous studies showing that IL-6 does play a role in LLD, but causality cannot be proven empirically. With respect to cognitive impairment, IL-6 was not verified to contribute in the non-depressive group, but an association was identified in the depressive group. LLD is a risk factor of dementia; thus, the outcomes merely prove that IL-6 is not a single or direct risk factor of cognitive impairment. Such cognitive impairment is induced by the reaction of IL-6 with other factors or via subsequent pathological issues such as depression.

### 5.3. Brain-Derived Neurotrophic Factor

Neurotrophins are a family of growth factors that modulate synaptic plasticity and neurotransmission, and there are four members of the neurotrophin family: nerve growth factor, BDNF, neurotrophin-3, and neurotrophin-4/5; among them, BDNF, with the widest distribution, is found mainly in the brain cortex and hippocampus [104,105]. BDNF has the function of protecting nerve death in the peripheral nerve system [106]; it is the main regulator of synaptic plasticity in the CNS, and it produces immediate and instructive regulation. BDNF gene is regulated by cell-type-specific and neuronal activity. In recent studies, we attempted to use technologies such as monoclonal BDNF antibody, an epitope-tagged BDNF knock-in mouse line (BDNF-Myc), and a conditional BDNF KO mouse line (cBDNF-ko) to detect where BDNF is manufactured and stored. Currently, there is evidence showing that BDNF exists in astrocytes, microglia, and postsynaptic dendrites, in addition to presynaptic axons and terminals [90,107]. The epitope-tagged BDNF knock-in mouse line (BDNF-HA) was found under immune-EM, and BDNF [108] was found in both presynaptic and postsynaptic vesicles of the hippocampus. The BDNF stored in postsynaptic vesicle was released under activity-dependent regulation [109]. In addition to nerve cells, BDNF [110] is found in both human platelets and megakaryocytes. The BDNF protein acts via the tropomycin receptor kinase (Trk) family of tyrosine kinase receptors. It is thought that BDNF/TrkB contribute to not only cognitive impairment (including learning deficits and memory impairment) but also mental disorders (including schizophrenia, mood disorders, intellectual disability, and autism) [111]. Malfunctioning BDNF/TrkB damages neural maintenance and regeneration, thus causing structural irregularities of brain. The BDNF gene features nine promoters that discretionarily generate BDNF transcripts, and its expression is regulated by calcium via Ca^2+^ influx, through Ca^2+^ permeable glutamate receptors (mainly *N*-methyl-d-aspartate (NMDA) receptors) and voltage-gated Ca^2+^ channels [65,112]. The BDNF protein can be edited epigenetically to yield various versions. For instance, a substitution single-nucleotide polymorphism (SNP) at codon 66 leads to the amino-acid valine being substituted by methionine (SNP rs6265), and this variant likely contributes to bipolar disorder [113,114]. Epigenetic phenomena refer to changes that affect gene activity and expression without changing the DNA sequence via DNA methylation and histone modifications. It has been found that the DNA methylation level of BDNF in the depression group exceeded that in the bipolar disorder and healthy groups [115,116]. Some animal trials showed that antidepressant treatment can reverse BDNF via its downregulation [117,118]. Ketamine, as a rapid-acting antidepressant, is probably associated with BDNF since it acts as follows: (1) increased phosphorylation and activation of TrkB [119]; (2) considerable release of BDNF at the hippocampus [120]; (3) activation of mammalian target of rapamycin (mTOR) signaling [51]. A 2 year longitudinal study by Diniz et al. (2014) [121] presented a comparative study of the BDNF levels in three groups: (1) remitted depression with incident mild cognitive impairment (LLD + MCI); (2) remitted depression without cognitive decline (LLD + NCD); (3) control group (no depression and no cognitive impairment). Additionally, a double-blind, placebo-controlled study by Diniz et al. (2014) [121] assessed the effects of donepezil treatment on the BDNF level. The study collected 158 samples from patients treated with the antidepressant; 130 with remitted depression treated with antidepressants later agreed to take part in the double-blind, placebo-controlled donepezil treatment trial. Among these 130 samples, there were 57 cases with incident mild cognitive impairment randomized to the donepezil or placebo group. At baseline and at months 12 and 24, the BDNF levels of all samples were tested. The findings showed that the BDNF level in the LLD + MCI and LLD + NCD groups presented no significant difference at baseline. However, in the 2 year trace, the BDNF level in all groups declined, whereas the donepezil treatment failed to alter the BDNF level. These outcomes seemingly demonstrate that the decline in BDNF levels was due to aging. By considering the unavoidability of the BDNF level dropping during aging, we can study its characteristics one factor at a time to determine any disparities in modulation and the influence of other factors.

## 6. Conclusions

Late-life depression (LLD), unlike early-onset depression (EOD), is a geriatric disease caused by a variety of factors. Serotonin is unlikely to effectively treat LLD, as it is thought to be associated with other biological factors. The glutamatergic system, inflammatory markers, and brain-derived neurotrophic factors may contribute to LLD and dementia. Ketamine, as a noncompetitive NMDAR antagonist, is the only officially approved clinical antidepressant; however, insufficient elderly were included in the pre-FDA-approved clinical trials. Therefore, a further clinical trial is needed to empirically prove the validity, safety, and reliability of ketamine as an antidepressant for LLD. The causality among cytokines, BDNF factors, and LLD remains uncertain, and it can likely be ascertained by applying anti-inflammatory agents or BDNF compounds in a clinical trial of the elderly. Clearly, disease arises from a physiological and mental interaction; thus, it is necessary to pay attention to cases of depression in the elderly, since improvements in physiological and psychiatric conditions may occur simultaneously.
